# Effects of breath-hold patterns, gadolinium concentrations and temporal resolutions on determination of mean left atrial circulation transit time by MR first pass perfusion

**DOI:** 10.1186/1532-429X-16-S1-P234

**Published:** 2014-01-16

**Authors:** Jie J Cao, Yi Wang, Michael Passick, Kathleen Bertman

**Affiliations:** 1 St Francis Hospital, Roslyn, New York, USA; 2 Cardiology, State University of New York at Stony Brook, Stony Brook, New York, USA

## Background

Normalized mean transit time in left atrium (nLATT) has potential to approximate left ventricular end diastolic pressure (LVEDP). In this study we prospectively evaluated the effects of gadolinium concentrations, breath-hold patterns and temporal resolutions on determination of nLATT by MR first pass perfusion.

## Methods

Twenty three patients were prospectively recruited to undergo MRI in a 1.5 T scanner. First pass perfusion imaging was performed using a saturation recovery steady state free precession sequence with ECG gating during breath-hold with Gadopentetate dimeglumine (Gd) injected at 0.01 mmol/kg. Imaging was repeated with free-breathing followed by breath-hold imaging using Gd at 0.025 mmol/kg separated with washout periods of 10 to 15 minutes. LATT was determined from the time intensity curves of the first pass using a custom program to assess time-signal integral and normalized by RR duration. nLATT was compared using paired t-test between images acquired in 3 settings. Additional 7 volunteers were recruited to undergo first pass perfusion with dynamic image acquired every 113 ms (TR) without ECG gating. To evaluate the effect of temporal resolution, LATT was assessed using simulated TR sampling dynamic signal intensity at multitudes of TR, respectively.

## Results

Of the 23 patients evaluated average nLATT was 8.60 ± 2.53 cardiac cycles (CC). Using published formula (LVEDP = 1.78 nLATT-3.78), mean LVEDP was estimated to be 12 ± 5 mmHg (ranging 5 to 24 mmHg). nLATT was shorter with free-breathing (7.57 ± 2.77 CC) resulting in lower LVEDP estimation of 10 ± 5 mmHg (p = 0.001). In contrast, nLATT was slightly prolonged (9.34 ± 3.36 CC) with higher Gd dosing resulting in modestly increased LVEDP estimation of 13 ± 6 mmHg (p = 0.106). In 7 patients tested with simulated TRs there was no significant change in LATT of 7.03 ± 1.6 s 7.02 ± 1.7 s, 7.38 ± 1.5 s, 7.07 ± 1.5 s, 7.66 ± 1.4 s and 7.59 ± 1.7 s corresponding to simulated TRs at 113 ms, 226 ms, 452 ms, 678 ms, 791 ms and 904 ms, respectively (p for trend = 0.406).

## Conclusions

While nLATT assessment using free-breathing acquisition may result in lower LVEDP estimation than breath-hold technique, albeit small absolute difference, the effects of Gd concentration (up to 0.025 mmol/kg) and temporal resolutions on nLATT assessment are limited.

## Funding

St Francis Research Fundation.

